# α–β–γ tracking filters using acceleration measurements

**DOI:** 10.1186/s40064-016-1960-8

**Published:** 2016-03-10

**Authors:** Kenshi Saho, Masao Masugi

**Affiliations:** Department of Electronic and Computer Engineering, Ritsumeikan University, Kusatsu, Shiga 525-8577 Japan

**Keywords:** α–β–γ filter, Moving target tracking, Acceleration measurements, Performance index, Optimal gain, Minimum variance filter criterion

## Abstract

**Background:**

Although real-time tracking of moving objects using a variety of sensor parameters is in great demand in monitoring systems, no studies have reported α–$\beta$–$\gamma$ tracking filters using simultaneous measurements including acceleration. In this report, we propose and analyze two α–$\beta$–$\gamma$ filters using acceleration measurements, namely, position–acceleration-measured (PAM) and position–velocity–acceleration-measured (PVAM) α–$\beta$–$\gamma$ filters.

**Findings:**

Based on our previous work on position–velocity-measured (PVM) α–$\beta$–$\gamma$ filters, performance indices of the proposed filters are theoretically derived. Then, numerical analyses clarify the conditions under which the performance of the PAM filter surpasses that of the position-only-measured (POM) α–$\beta$–$\gamma$ filter. The results indicate that the PVAM filter achieves better accuracy than the other filters, even with a relatively large measurement noise.

**Conclusions:**

This report verifies the effectiveness of the $\alpha$–$\beta$–$\gamma$ filters using acceleration measurements based on numerical analyses using derived performance indices. These results are useful in the design of tracking systems including acceleration measurements (e.g., in deciding whether to use the measured acceleration to improve tracking filter performance).

**Electronic supplementary material:**

The online version of this article (doi:10.1186/s40064-016-1960-8) contains supplementary material, which is available to authorized users.

## Background

Real-time tracking of moving objects is an essential function in intelligent vehicles. The most commonly used approaches to this task involve the use of Kalman tracking filters and their extensions (e.g., particle filters and interacting-multiple-model filters) (Du and Zhang 2015; Jin et al. 2015). However, these techniques have high computational loads, which thus render them unsuitable for many applications. In contrast, alpha–beta–gamma filters do not suffer from these applicability problems because they involve only small computational loads (Tenne and Singh 2002). For modern sensors that can also measure velocity we previously proposed position–velocity-measured (PVM) $\alpha$–$\beta$–$\gamma$ filters and verified their effectiveness analytically compared with the widely used Kalman filter and position-only-measured (POM) $\alpha$–$\beta$–$\gamma$ filter (Saho and Masugi 2015). Based on this study, it is feasible that $\alpha$–$\beta$–$\gamma$ filtering can be effectively implemented for various velocity-measured sensors such as micro-Doppler radars (Kozma et al. 2012; Lim et al. 2010). However, recent technological improvements in the Internet of Things have facilitated the development of sensing systems that can measure various parameters besides just position and velocity. For tracking systems, acceleration is the most valuable of these. However, tracking filters using simultaneous position, velocity, and acceleration measurements have only been considered in a small number of studies (Besada et al. 2007; Lau et al. 2013; Lefas 1984). Furthermore, no study has focused on $\alpha$–$\beta$–$\gamma$ filters assuming simultaneous measurements including acceleration.

In this report, we analyze $\alpha$–$\beta$–$\gamma$ filters by extending our previous work (Saho and Masugi 2015) to acceleration measurements. Two acceleration-measured $\alpha$–$\beta$–$\gamma$ filters are proposed: a position–acceleration-measured (PAM) $\alpha$–$\beta$–$\gamma$ filter and a position–velocity–acceleration-measured (PVAM) $\alpha$–$\beta$–$\gamma$ filter. We derive performance indices analytically and calculate the optimal gain using a minimum-variance (MV) filter criterion (Kosuge and Ito 2003). Numerical analyses verify the effectiveness of the proposed filters compared with the conventional POM and PVM filters.

## Proposed acceleration-measured* α*–*β*–*γ* filters

### Position–acceleration-measured* α*–*β*–*γ* filter

The $\alpha$–$\beta$–$\gamma$ filter predicts the position, velocity, and acceleration of a moving target based on a constant acceleration model using the gains from three filters (Tenne and Singh 2002). This filter iterates the prediction and smoothing processes. The prediction process is expressed as follows:

1 $$x_{{\mathrm{p}}k} = x_{{\mathrm{s}}k-1}+Tv_{{\mathrm{s}}k-1}+(T^2/2)a_{{\mathrm{s}}k-1},$$

2 $$v_{{\mathrm{p}}k} = v_{{\mathrm{s}}k-1}+Ta_{{\mathrm{s}}k-1},$$

3 $$a_{{\mathrm{p}}k} = a_{{\mathrm{s}}k-1},$$

where *T* is the sampling interval, $x_{{\mathrm{s}}k}$ and $x_{\mathrm{p}k}$ are, respectively, the smoothed and predicted target position at time *kT*, $v_{{\mathrm{s}}k}$ and $v_{{\mathrm{p}}k}$ are, respectively, the smoothed and predicted target velocities, and $a_{{\mathrm{s}}k}$ and $a_{{\mathrm{p}}k}$ are, respectively, the smoothed and predicted target acceleration. The prediction process is common in all $\alpha$–$\beta$–$\gamma$ filters.

We define the smoothing process for a new $\alpha$–$\beta$–$\gamma$ filter using the measured position and acceleration as:

4 $$x_{{\mathrm{s}}k} = x_{{\mathrm{p}}k}+\alpha (x_{{\mathrm{o}}k}-x_{{\mathrm{p}}k}),$$

5 $$v_{{\mathrm{s}}k} = v_{{\mathrm{p}}k}+(\beta /T)(x_{{\mathrm{o}}k}-x_{{\mathrm{p}}k}),$$

6 $$a_{{\mathrm{s}}k} = a_{{\mathrm{p}}k}+\gamma (a_{{\mathrm{o}}k}-a_{{\mathrm{p}}k}),$$

where $x_{{\mathrm{o}}k}$ is the measured position, $a_{{\mathrm{o}}k}$ is the measured acceleration, and $\alpha$, $\beta$, and $\gamma$ are the filter gains. We call the $\alpha$–$\beta$–$\gamma$ filter defined by (1)–(3) and (4)–(6), the PAM filter. This filter differs from the widely used POM filter in that (6) uses the measured acceleration instead of the measured position.

### Position–velocity–acceleration-measured α–β–γ filter

If the position, velocity, and acceleration are all observable, we can realize more accurate tracking than is possible with the PVM and PAM filters. We therefore, propose the PVAM $\alpha$–$\beta$–$\gamma$ filter in which the smoothing process is defined as:

7 $$x_{{\mathrm{s}}k} = x_{{\mathrm{p}}k}+\alpha (x_{{\mathrm{o}}k}-x_{{\mathrm{p}}k}),$$

8 $$v_{{\mathrm{s}}k} = v_{{\mathrm{p}}k}+\beta (v_{{\mathrm{o}}k}-v_{{\mathrm{p}}k}),$$

9 $$a_{{\mathrm{s}}k} = a_{{\mathrm{p}}k}+\gamma (a_{{\mathrm{o}}k}-a_{{\mathrm{p}}k}),$$

where $v_{{\mathrm{o}}k}$ is the measured velocity. We call the $\alpha$–$\beta$–$\gamma$ filter defined by (1)–(3) and (7)–(9), the PVAM filter. This filter differs from the PVM filters (Saho and Masugi 2015) in that (9) uses the measured acceleration instead of the measured position or velocity. If both the velocity and acceleration measurements are sufficiently accurate, the proposed PVAM filter can track substantially more accurately than the other $\alpha$–$\beta$–$\gamma$ filters.

## Performance indices and optimal gain

Two efficient performance indices of the $\alpha$–$\beta$–$\gamma$ filters and their derivation results are presented here. We also explain the design of an optimal gain method based on these indices, which is known as the minimum-variance (MV) filter criterion (Kosuge and Ito 2003).

### Smoothing performance index

A reduction in random measurement errors is a fundamental function of a tracking filter. One indicator of the performance in this regard is the steady-state error of a target under constant acceleration considering measurement noise. The variance in the predicted target position in the steady state is calculated by (Tenne and Singh 2002):

10 $$\begin{aligned} \sigma _{\mathrm{p}}^2=E\left[ (x_{{\mathrm{p}}k}-x_{{\mathrm{t}}k})^2\right] , \end{aligned}$$

where $x_{{\mathrm{t}}k}$ is the true target position and *E*[ ] indicates the mean. $\sigma _{\mathrm{p}}^2$ is called the smoothing performance index.

We now show the derivation results of the smoothing performance indices of the proposed filters. The derivation procedure for these is the same as that for the PVM filters presented in (Saho and Masugi 2015) and is given in the Additional file 1. $\sigma _{\mathrm{p}}^2$ of the PAM filter is derived from (1)–(3) and (4)–(6) as:

11 $$\begin{aligned} \sigma _{\mathrm{p, PAM}}^2&= \frac{2\alpha ^2+2\beta +\alpha \beta }{\alpha (4-2\alpha -\beta )}B_{\mathrm{x}} \nonumber \\&+\, \frac{\gamma (\alpha +\gamma -\alpha \gamma )}{2\alpha \beta (\alpha \gamma ^2-\gamma ^2-\alpha \gamma +\beta \gamma - \beta )}T^4B_{\mathrm{a}}, \nonumber \\ \end{aligned}$$

where $B_{\mathrm{x}}$ and $B_{\mathrm{a}}$ are the variances in the white Gaussian noise in $x_{{\mathrm{o}}k}$ and $a_{{\mathrm{o}}k}$, respectively.

The smoothing performance index of the PVAM filter is similarly derived as:

12 $$\begin{aligned} \sigma _{\mathrm{p, PVAM}}^2 &= \frac{\alpha }{2-\alpha }B_{\mathrm{x}} \nonumber \\ &\quad +\,\frac{\beta (2-\alpha -\beta +\alpha \beta )}{\alpha (2-\alpha )(2-\beta )(\alpha +\beta -\alpha \beta )}T^2B_{\mathrm{v}} \nonumber \\&\quad +\,\gamma \frac{h_1(\alpha , \beta , \gamma )}{h_2(\alpha , \beta , \gamma )}T^4B_{\mathrm{a}}, \end{aligned}$$

where $B_{\mathrm{v}}$ is the variance in the white Gaussian noise in $v_{{\mathrm{o}}k}$ and

13 $$\begin{aligned} h_1(\alpha , \beta , \gamma ) &= \gamma ^2(1-\alpha )(1-\beta )(13\alpha \beta ^3 \nonumber \\ &\quad -\,13\beta ^3-39 \alpha \beta ^2+54 \beta ^2 \nonumber \\&\quad +\,38 \alpha \beta -68 \beta -12 \alpha +24) \nonumber \\&\quad -\,\gamma (26 \alpha ^2 \beta ^4-45 \alpha \beta ^4 \nonumber \\&\quad +\,19 \beta ^4-95 \alpha ^2 \beta ^3+192 \alpha \beta ^3\nonumber \\&\quad -\,88 \beta ^3+127 \alpha ^2 \beta ^2-290 \alpha \beta ^2 \nonumber \\ &\quad +\,136 \beta ^2-78 \alpha ^2 \beta +188 \alpha \beta \nonumber \\&\quad -\,80 \beta +20 \alpha ^2-48 \alpha +16) \nonumber \\&\quad +\,13 \alpha ^2 \beta ^4-19 \alpha \beta ^4+6 \beta ^4 \nonumber \\&\quad -\,43 \alpha ^2 \beta ^3+80 \alpha \beta ^3-28 \beta ^3 \nonumber \\&\quad +\,50 \alpha ^2 \beta ^2-112 \alpha \beta ^2+40 \beta ^2 \nonumber \\&\quad -\,28 \alpha ^2 \beta +64 \alpha \beta -16 \beta \nonumber \\&\quad +\,8 \alpha ^2-16 \alpha , \end{aligned}$$

14 $$\begin{aligned} h_2(\alpha , \beta , \gamma )&= 4\alpha \beta (2-\alpha )(2-\beta )(2-\gamma ) \nonumber \\&\cdot \,(\alpha \beta -\alpha -\beta ) \nonumber \\&\cdot \,(\alpha \gamma -\alpha -\gamma ) \nonumber \\&\cdot \,(\beta \gamma -\beta -\gamma ). \end{aligned}$$

### Tracking performance index

The tracking filter is required to track complicated motion, including jerks. The steady-state bias error incurred when tracking a target moving with constant jerk is used to evaluate the performance of the $\alpha$–$\beta$–$\gamma$ filter and is calculated as (Kosuge and Ito 2001)

15 $$\begin{aligned} e_{\mathrm{fin}}=\lim _{k \rightarrow \infty } \{J(kT)^3/6-x_{{\mathrm{p}}k} \}, \end{aligned}$$

where *J* is the constant jerk of the target and $J(kT)^3/6$ is the true position of a target moving with constant jerk. $e_{\mathrm{fin}}$ is called the tracking performance index.

The tracking performance indices of the PAM and PVAM filters can then be derived as

16 $$\begin{aligned} e_{\mathrm{fin, PAM}}= \frac{2-\gamma }{2\beta \gamma }JT^3, \end{aligned}$$

and

17 $$\begin{aligned} e_{\mathrm{fin, PVAM}}=\frac{6-3\beta -3\gamma +\beta \gamma }{6\alpha \beta \gamma }JT^3. \end{aligned}$$

The derivations of (12) and (17) are also given in the Additional file 1. The smaller these tracking/smoothing performance indices are, the better is the tracking filter. However, there is a trade-off between $e_{\mathrm{fin}}$ and $\sigma _{\mathrm{p}}^2$.

### Optimal gain determination with MV filter criterion

The MV filter criterion has been proposed as a method for determining the optimal gain using the performance indices given above and considering the trade-off between these indices (Kosuge and Ito 2003). The effectiveness of this criterion for PVM filters is discussed in our previous work (Saho and Masugi 2015). This criterion determines the gain by minimizing $\sigma _{\mathrm{p}}^2$ while keeping $e_{\mathrm{fin}}$ constant. The optimal gain with the MV filter criterion is determined by:

18 $$\begin{aligned}&\arg \min _{\alpha , \beta } \; \sigma _{\mathrm{p}}^2 \nonumber \\&{\mathrm{sub.}} \; {\mathrm{to}} \; \; \; e_{\mathrm{fin}} = {\mathrm{const.}} \end{aligned}$$

Solving (18) with (11) and (16) determines the optimal gain of the PAM filter. Similarly, solving (18) with (12) and (17) determines the optimal gain of the PVAM filter. These are solved sufficiently quickly using the simple gradient descent method. Using the optimal gains, optimal smoothing performance index $\sigma ^2_{\mathrm{p, opt}}$ is calculated for each $e_{\mathrm{fin}}$.

## Analysis results and discussion

We evaluated the performance of the proposed $\alpha$–$\beta$–$\gamma$ filters analytically with optimal gain calculated by the MV filter criterion, and compared it with the POM and PVM filters. We clarify the relationship between the measurement noise and filter performance. The following analyses assume that *T*, $B_{\mathrm{x}}$, and *J* are normalized to 1 and, for this discussion, use measurement noise defined as:

19 $$\begin{aligned} R_{\mathrm{a}} = T^4B_{\mathrm{a}}/B_{\mathrm{x}}. \end{aligned}$$

20 $$\begin{aligned} R_{\mathrm{v}} = T^2B_{\mathrm{v}}/B_{\mathrm{x}}. \end{aligned}$$

Note that there are two types of PVM filters: A-V (acceleration smoothed by measured velocity) and A-P (acceleration smoothed by measured position) filters (see Saho and Masugi 2015), which are referred to in this section as PVM-AV and PVM-AP filters, respectively.

**Fig. 1:**
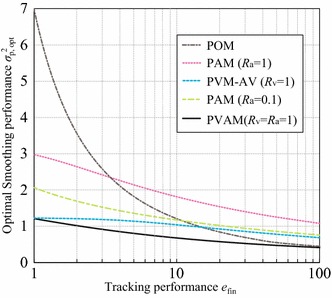
Relationship between $e_{\mathrm{fin}}$ and $\sigma ^2_{\mathrm{p, opt}}$ of various filters

**Fig. 2:**
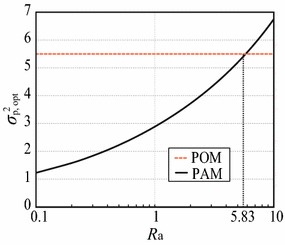
Relationship between $R_{\mathrm{a}}$ and $\sigma ^2_{\mathrm{p, opt}}$ of PAM and POM filters for $\Gamma =1.25$

**Fig. 3:**
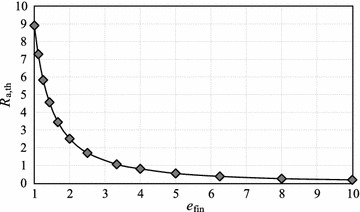
Relationship between $e_{\mathrm{fin}}$ and $R_{\mathrm{a, th}}$

**Fig. 4:**
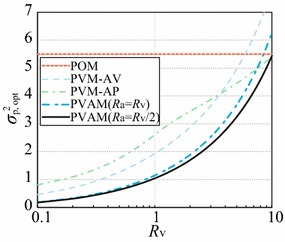
Relationship between $R_{\mathrm{a}}$ and optimal $\sigma ^2_{\mathrm{p, opt}}$ of PVAM, PVM, and POM filters for $e_{\mathrm{fin}}=1.25$

### Relationship between performance indices

Figure 1 shows the smoothing performance index $\sigma ^2_{\mathrm{p, opt}}$ obtained using the optimal gain for each tracking performance index $e_{\mathrm{fin}}$. The performance difference between filters increases for relatively small $e_{\mathrm{fin}}$. First, we compared the performance of the POM and PAM filters assuming that only position and acceleration are measured. When $e_{\mathrm{fin}}=1$, the value of $\sigma ^2_{\mathrm{p,opt}}$ for the PAM filter with $R_{\mathrm{a}}=1$ is 3/7 of that for the POM filter. In contrast, when approximately $e_{\mathrm{fin}}>3.3$, the performance of the PAM filter is worse than that of the POM filter. Naturally, better performance is achieved with better acceleration measurement accuracy as shown by the result of the PAM filter with $R_{\mathrm{a}}=0.1$. These results indicate that the proposed PAM filter yields improved performance compared with the POM filter by using sufficiently accurate acceleration measurements.

We also compared the performance of the PVAM, PVM, and PAM filters. The PVAM filter results shown in Fig. 1 indicate improvements over the PVM and PAM filters due to the use of measured acceleration. The value of $\sigma ^2_{\mathrm{p, opt}}$ for the PVAM filter is smaller than that of the PVM and PAM filters for all $e_{\mathrm{fin}}$, even when $R_{\mathrm{v}}=R_{\mathrm{a}}=1$. Note that when the position, velocity, and acceleration measurements are equally reliable ($R_{\mathrm{v}}=R_{\mathrm{a}}=1$), the PVAM filter achieves the best performance, followed in order by the PVM, PAM, and POM filters for relatively small $e_{\mathrm{fin}}$. This is an important consideration in designing real tracking systems.

### Relationship between $\sigma ^2_{\mathrm{p, opt}}$ and the measurement noise ratio ($R_{\mathrm{a}}$ and $R_{\mathrm{v}}$)

We clarify the relationship between the measurement noise ratio and the smoothing performance index $\sigma ^2_{\mathrm{p,opt}}$. Figure 2 shows $\sigma ^2_{\mathrm{p, opt}}$ for the POM and PAM filters as a function of $R_{\mathrm{a}}$ for $e_{\mathrm{fin}}=1.25$. This figure clearly demonstrates that we must use the measured acceleration for $R_{\mathrm{a}}<5.83$. Otherwise, we must select a POM filter to avoid performance deterioration resulting from the use of inaccurately measured acceleration. This $R_{\mathrm{a}}$ threshold is useful in deciding whether to use the measured acceleration. Hence, we now define $R_{\mathrm{a, th}}$, which is the $R_{\mathrm{a}}$ value for which the $\sigma ^2_{\mathrm{p,opt}}$ values for the PAM and POM filters are equal. This means that the smoothing performance of the PAM filter is better than that of the POM filter when $R_{\mathrm{a}}<R_\mathrm{a,th}$ (as indicated above, $R_{\mathrm{a,th}}=5.83$ for $e_{\mathrm{fin}}=1.25$). Figure 3, which shows the relationship between $e_{\mathrm{fin}}$ and $R_{\mathrm{a,th}}$, clearly indicates when we should and should not use the measured acceleration. The curve in Fig. 3 can be expressed by power approximation as

21 $$\begin{aligned} R_{\mathrm{a,th}}(e_{\mathrm{fin}})=8.3926 \, e_{\mathrm{fin}}^{-1.686}, \end{aligned}$$

where its coefficient of determination is equal to 0.999. This equation is most useful in the design of tracking systems using position and acceleration measurements.

The relationships between the $\sigma ^2_{\mathrm{p, opt}}$ values of the POM, PVM, and PVAM filters and $R_{\mathrm{v}}$ are also investigated and shown in Fig. 4. Clearly, the PVAM filter achieves better accuracy than the PVM-AV filter when $R_{\mathrm{a}}=R_{\mathrm{v}}$. Moreover, the PVAM filter with $R_{\mathrm{a}}=R_{\mathrm{v}}/2$ achieves better performance than the PVM-AP filter, even for relatively large $R_{\mathrm{v}}$. These results verify theoretically that the proposed PAM and PVAM filters succeed in improving the tracking accuracy of the conventional POM and PVM filters.

## Conclusion

We proposed two $\alpha$–$\beta$–$\gamma$ filters: the PAM and PVAM filters. The derivation results of the performance indices of these filters are shown as (11), (12), (16), (17). Numerical analyses showed that the performance of the PAM filter is better than that of the POM filter when $R_{\mathrm{a}}<R_{\mathrm{a, th}}(e_{\mathrm{fin}})$ as indicated in (21). This is useful in the design of tracking systems using position/acceleration measurements. The results also show that the PVAM filter achieves the best performance even when $R_{\mathrm{v}}=R_{\mathrm{a}}=1$.

## Additional file

10.1186/s40064-016-1960-8 Derivation of the performance indices of the proposed PAM and PVAM filters Derivation of (11), (12), (16), and (17) are given in this additional file.

## List of abbreviations

MV: minimum-variance
PAM filter: position–acceleration-measured α–β–γ filter
POM filter: position-only-measured α–β–γ filter
PVAM filter: position–velocity–acceleration-measured α–β–γ filter
PVM filter: position–velocity-measured α–β–γ filter
PVM-AP filter: PVM acceleration smoothed by measured position-type α–β–γ filter
PVM-AV filter: PVM acceleration smoothed by measured velocity-type α–β–γ filter
