# Supplementary material for: α–β–γ tracking filters using acceleration measurements
Source: Springerplus. 2016 Mar 10;5:309. doi: 10.1186/s40064-016-1960-8 (PMC4786520; doi:10.1186/s40064-016-1960-8)
Supplement: Supplementary file 1 — 10.1186/s40064-016-1960-8 Derivation of the performance indices of the proposed PAM and PVAM filters Derivation of (11), (12), (16), and (17) are given in this additional file. [file 40064_2016_1960_MOESM1_ESM.pdf]

Derivations of the tracking and smoothing performance indices of the proposed PAM and PVAM filters

Introduction

In this additional documentation, we give the derivations of the tracking and smoothing performance indices of the proposed PAM and PVAM filters ((11), (12), (16), and (17) of the main document).

Definitions

The PAM filter is defined by (1)–(6) of the main document as:

$$\begin{aligned} x_{\mathrm{p}k} &= x_{\mathrm{s}k-1} + Tv_{\mathrm{s}k-1} + (T^2/2)a_{\mathrm{s}k-1}, && (1) \\ v_{\mathrm{p}k} &= v_{\mathrm{s}k-1} + Ta_{\mathrm{s}k-1}, && (2) \\ a_{\mathrm{p}k} &= a_{\mathrm{s}k-1}, && (3) \\ x_{\mathrm{s}k} &= x_{\mathrm{p}k} + \alpha(x_{\mathrm{o}k} - x_{\mathrm{p}k}), && (4) \\ v_{\mathrm{s}k} &= v_{\mathrm{p}k} + (\beta/T)(x_{\mathrm{o}k} - x_{\mathrm{p}k}), && (5) \\ a_{\mathrm{s}k} &= a_{\mathrm{p}k} + \gamma(a_{\mathrm{o}k} - a_{\mathrm{p}k}). && (6) \end{aligned}$$

where $x_{\mathrm{s}k}$ is the smoothed target position at time kT, T is the sampling interval, $x_{\mathrm{p}k}$ is the predicted target position, $v_{\mathrm{s}k}$ is the smoothed target velocity, $v_{\mathrm{p}k}$ is the predicted target velocity, $a_{\mathrm{s}k}$ is the smoothed target acceleration, $a_{\mathrm{p}k}$ is the predicted target acceleration, $x_{\mathrm{o}k}$ is the measured position, $a_{\mathrm{o}k}$ is the measured acceleration, and α, β, and γ are the filter gains. The PVAM filter is defined by (1)–(3) and (7)–(9) of the main document. The smoothing process of the PVAM filter are:

$$\begin{aligned} x_{\mathrm{s}k} &= x_{\mathrm{p}k} + \alpha(x_{\mathrm{o}k} - x_{\mathrm{p}k}), && (7) \\ v_{\mathrm{s}k} &= v_{\mathrm{p}k} + \beta(v_{\mathrm{o}k} - v_{\mathrm{p}k}), && (8) \\ a_{\mathrm{s}k} &= a_{\mathrm{p}k} + \gamma(a_{\mathrm{o}k} - a_{\mathrm{p}k}), && (9) \end{aligned}$$

where $v_{\mathrm{o}k}$ is the measured velocity. The smoothing and tracking performance indices σ_{p}^2 and e_{fin} are defined as (10) and (15) of the main document, that are:

$$\begin{aligned} \sigma_{\mathrm{p}}^2 &= E[(x_{\mathrm{p}k} - x_{\mathrm{t}k})^2], && (10) \\ e_{\mathrm{fin}} &= \lim_{k\to\infty} \{J(kT)^3/6 - x_{\mathrm{p}k}\}, && (11) \end{aligned}$$

where $x_{\mathrm{t}k}$ is the true target position, $E[\,]$ indicates the mean, and J is the constant jerk of the target.

Derivation of $\sigma^2_{\mathrm{p,PAM}}$

The true position of a target moving with constant acceleration is

$$x_{\mathrm{t}k} = x_{\mathrm{t}k-1} + Tv_{\mathrm{t}k-1} + (T^2/2)a_{\mathrm{t}k-1}, \quad (12)$$

where v_{t} and a_{t} are the true velocity and acceleration. With (1) and (12), the variance of the predicted position errors is

$$\begin{aligned}\sigma^2_{\mathrm{p}} &= E[(x_{\mathrm{p}k} - x_{\mathrm{t}k})^2] \\ &= E[(x_{\mathrm{s}k-1} - x_{\mathrm{t}k-1})^2] + T^2E[(v_{\mathrm{s}k-1} - v_{\mathrm{t}k-1})^2] \\ &\quad +(T^4/4)E[(a_{\mathrm{s}k-1} - a_{\mathrm{t}k-1})^2] + 2TE[(x_{\mathrm{s}k-1} - x_{\mathrm{t}k-1})(v_{\mathrm{s}k-1} - v_{\mathrm{t}k-1})] \\ &\quad +T^2E[(x_{\mathrm{s}k-1} - x_{\mathrm{t}k-1})(a_{\mathrm{s}k-1} - a_{\mathrm{t}k-1})] \\ &\quad +T^3E[(v_{\mathrm{s}k-1} - v_{\mathrm{t}k-1})(a_{\mathrm{s}k-1} - a_{\mathrm{t}k-1})]. \end{aligned} \quad (13)$$

Because we assume a steady state, the variances and covariances in (13) do not depend on k. Consequently, we can define these variances and covariances as:

$$\begin{aligned}\sigma^2_{\mathrm{sx}} &= E[(x_{\mathrm{s}k} - x_{\mathrm{t}k})^2] = E[(x_{\mathrm{s}k-1} - x_{\mathrm{t}k-1})^2], &(14)\\ \sigma^2_{\mathrm{sv}} &= E[(v_{\mathrm{s}k} - v_{\mathrm{t}k})^2] = E[(v_{\mathrm{s}k-1} - v_{\mathrm{t}k-1})^2], &(15)\\ \sigma^2_{\mathrm{sa}} &= E[(a_{\mathrm{s}k} - a_{\mathrm{t}k})^2] = E[(a_{\mathrm{s}k-1} - a_{\mathrm{t}k-1})^2], &(16)\\ \sigma^2_{\mathrm{sxv}} &= E[(x_{\mathrm{s}k} - x_{\mathrm{t}k})(v_{\mathrm{s}k} - v_{\mathrm{t}k})] = E[(x_{\mathrm{s}k-1} - x_{\mathrm{t}k-1})(v_{\mathrm{s}k-1} - v_{\mathrm{t}k-1})], &(17)\\ \sigma^2_{\mathrm{sxa}} &= E[(x_{\mathrm{s}k} - x_{\mathrm{t}k})(a_{\mathrm{s}k} - a_{\mathrm{t}k})] = E[(x_{\mathrm{s}k-1} - x_{\mathrm{t}k-1})(a_{\mathrm{s}k-1} - a_{\mathrm{t}k-1})], &(18)\\ \sigma^2_{\mathrm{sva}} &= E[(v_{\mathrm{s}k} - v_{\mathrm{t}k})(a_{\mathrm{s}k} - a_{\mathrm{t}k})] = E[(v_{\mathrm{s}k-1} - v_{\mathrm{t}k-1})(a_{\mathrm{s}k-1} - a_{\mathrm{t}k-1})]. &(19)\end{aligned}$$

Substituting (14)–(19) into (13), we have

$$\sigma^2_{\mathrm{p}} = \sigma^2_{\mathrm{sx}} + T^2\sigma^2_{\mathrm{sv}} + (T^4/4)\sigma^2_{\mathrm{sa}} + 2T\sigma^2_{\mathrm{sxv}} + T^2\sigma^2_{\mathrm{sxa}} + T^3\sigma^2_{\mathrm{sva}}. \quad (20)$$

The variances and covariances in this equation are derived as functions of the filter gains and the measurement noise variances. With (1) and (4), we have

$$x_{\mathrm{s}k} = (1-\alpha)(x_{\mathrm{s}k-1} + Tv_{\mathrm{s}k-1} + (T^2/2)a_{\mathrm{s}k-1}) + \alpha x_{\mathrm{o}k}. \quad (21)$$

We can rewrite (12) as

$$x_{\mathrm{t}k} = (1-\alpha)(x_{\mathrm{t}k-1} + Tv_{\mathrm{t}k-1} + (T^2/2)a_{\mathrm{t}k-1}) + \alpha x_{\mathrm{t}k}. \quad (22)$$

Using (21) and (22), the smoothing error is expressed as

$$\begin{aligned}x_{\mathrm{s}k} - x_{\mathrm{t}k} &= (1-\alpha)\{(x_{\mathrm{s}k-1} - x_{\mathrm{t}k-1}) + T(v_{\mathrm{s}k-1} - v_{\mathrm{t}k-1}) \\ &\quad +(T^2/2)(a_{\mathrm{s}k-1} - a_{\mathrm{t}k-1})\} + \alpha(x_{\mathrm{o}k} - x_{\mathrm{t}k}).\end{aligned} \quad (23)$$

Thus, the variance of this error is calculated as

$$\sigma^2_{\mathrm{sx}} = E[(x_{\mathrm{s}k} - x_{\mathrm{t}k})^2]$$

$$
\begin{aligned}
= \quad & (1-\alpha)^2\{E[(x_{\mathrm{s}k-1}-x_{\mathrm{t}k-1})^2]+T^2E[(v_{\mathrm{s}k-1}-v_{\mathrm{t}k-1})^2] \\
& +(T^4/4)E[(a_{\mathrm{s}k-1}-a_{\mathrm{t}k-1})^2]+2TE[(x_{\mathrm{s}k-1}-x_{\mathrm{t}k-1})(v_{\mathrm{s}k-1}-v_{\mathrm{t}k-1})] \\
& +T^2E[(x_{\mathrm{s}k-1}-x_{\mathrm{t}k-1})(a_{\mathrm{s}k-1}-a_{\mathrm{t}k-1})] \\
& +T^3E[(v_{\mathrm{s}k-1}-v_{\mathrm{t}k-1})(a_{\mathrm{s}k-1}-a_{\mathrm{t}k-1})]\}+\alpha^2E[(x_{\mathrm{o}k}-x_{\mathrm{t}k})^2] \\
& +2\alpha(1-\alpha)\{E[(x_{\mathrm{s}k-1}-x_{\mathrm{t}k-1})(x_{\mathrm{o}k}-x_{\mathrm{t}k})] \\
& +TE[(v_{\mathrm{s}k-1}-v_{\mathrm{t}k-1})(x_{\mathrm{o}k}-x_{\mathrm{t}k})] \\
& +(T^2/2)E[(a_{\mathrm{s}k-1}-a_{\mathrm{t}k-1})(x_{\mathrm{o}k}-x_{\mathrm{t}k})]\}. \qquad (24)
\end{aligned}
$$

Here,

$$E[(x_{\mathrm{o}k}-x_{\mathrm{t}k})^2]=B_{\mathrm{x}}. \qquad (25)$$

The following relations are satisfied because of the steady-state assumption and because the smoothed parameters are a linear combination of the measured parameters:

$$
\begin{aligned}
E[(x_{\mathrm{s}k-1}-x_{\mathrm{t}k-1})(x_{\mathrm{o}k}-x_{\mathrm{t}k})] &= 0, & (26)\\
E[(v_{\mathrm{s}k-1}-v_{\mathrm{t}k-1})(x_{\mathrm{o}k}-x_{\mathrm{t}k})] &= 0, & (27)\\
E[(a_{\mathrm{s}k-1}-a_{\mathrm{t}k-1})(x_{\mathrm{o}k}-x_{\mathrm{t}k})] &= 0. & (28)
\end{aligned}
$$

Substituting (14)–(19) and (25)–(28) into (24), we obtain

$$
\begin{aligned}
\sigma_{\mathrm{sx}}^2 \quad = \quad & (1-\alpha)^2(\sigma_{\mathrm{sx}}^2+T^2\sigma_{\mathrm{sv}}^2+(T^4/4)\sigma_{\mathrm{sa}}^2+2T\sigma_{\mathrm{sxv}}^2+T^2\sigma_{\mathrm{sxa}}^2+T^3\sigma_{\mathrm{sva}}^2) \\
& +\alpha^2B_{\mathrm{x}}. \qquad (29)
\end{aligned}
$$

This can be simplified to

$$
\begin{aligned}
& \alpha(2-\alpha)\sigma_{\mathrm{sx}}^2-(1-\alpha)^2(T^2\sigma_{\mathrm{sv}}^2+(T^4/4)\sigma_{\mathrm{sa}}^2+2T\sigma_{\mathrm{sxv}}^2+T^2\sigma_{\mathrm{sxa}}^2+T^3\sigma_{\mathrm{sva}}^2) \\
& =\alpha^2B_{\mathrm{x}}. \qquad (30)
\end{aligned}
$$

In the same way, other variances and covariances are calculated using (1)–(3) and (4)–(6), and their simplification yields:

$$
\begin{aligned}
& \beta^2\sigma_{\mathrm{sx}}^2+\beta(2-\beta)T^2\sigma_{\mathrm{sv}}^2+(1-\beta/2)^2T^4\sigma_{\mathrm{sa}}^2+2\beta(\beta-1)T\sigma_{\mathrm{sxv}}^2 \\
& +(1-\beta)(2-\beta)T^3\sigma_{\mathrm{sva}}^2\ +(\beta-2)T^2\sigma_{\mathrm{sxa}}^2\ =-\beta^2B_{\mathrm{x}}, \qquad (31)\\
& \gamma(2-\gamma)\sigma_{\mathrm{sa}}^2=\gamma^2B_{\mathrm{a}}, \qquad (32)\\
& \beta(\alpha-1)\sigma_{\mathrm{sx}}^2+(1-\alpha)(1-\beta)T^2\sigma_{\mathrm{sv}}^2+(1-\alpha)(1-\beta/2)/2T^4\sigma_{\mathrm{sa}}^2 \\
& +(2\alpha\beta-\alpha-2\beta)T\sigma_{\mathrm{sxv}}^2\ +(\alpha-1)(\beta-1)T^3\sigma_{\mathrm{sva}}^2+(\alpha-1)(2\beta-3)/2T^2\sigma_{\mathrm{sxa}}^2 \\
& =-\alpha\beta B_{\mathrm{x}}, \qquad (33)\\
& (1-\alpha)(1-\gamma)/2T^2\sigma_{\mathrm{sa}}^2+(\alpha\gamma-\alpha-\gamma)\sigma_{\mathrm{sxa}}^2+(1-\alpha)(1-\gamma)T\sigma_{\mathrm{sva}}^2=0, \qquad (34)\\
& (1-\beta/2)(1-\gamma)/2T^2\sigma_{\mathrm{sa}}^2+\beta(\gamma-1)\sigma_{\mathrm{sxa}}^2+(\beta\gamma-\beta-\gamma)T\sigma_{\mathrm{sva}}^2=0, \qquad (35)
\end{aligned}
$$

where

$$E[(a_{ok}-a_{tk})^2]=B_a, \tag{36}$$

Also, the following is satisfied because we assume that the position and acceleration measurement noise are uncorrelated:

$$E[(x_{ok}-x_{tk})(a_{ok}-a_{tk})]=0. \tag{37}$$

Solving the linear system involving (30)–(35) using the Cramer's rule, and substituting the solutions into (20), we have $\sigma_{\mathrm{p,PAM}}$ ((11) of the main document).

Derivation of $e_{\mathrm{fin,PAM}}$

We first derive the relationship between the measured signals (x_{ok} and a_{ok}) and x_{pk} in the z-domain, and then obtain the tracking performance index using the final value theorem. Applying a z-transform to (1)–(3) and (4)–(6), we obtain:

$$\begin{aligned}
X_p(z) &= X_s(z)/z+TV_s(z)/z+(T^2/2)A_s(z)/z, && (38)\\
V_p(z) &= V_s(z)/z+TA_s(z)/z, && (39)\\
A_p(z) &= A_s(z)/z, && (40)\\
X_s(z) &= X_p(z)+\alpha(X_o(z)-X_p(z)), && (41)\\
V_s(z) &= V_p(z)+(\beta/T)(X_o(z)-X_p(z)), && (42)\\
A_s(z) &= A_p(z)+\gamma(A_o(z)-A_p(z)), && (43)
\end{aligned}$$

where uppercase letters indicate the z-transform of the variables expressed in lowercase letters. Substituting (40) into (43) gives

$$A_p(z) = \frac{\gamma}{z+\gamma-1}A_o(z). \tag{44}$$

With (39), (40), and (42), we have

$$(z-1)V_p(z) = (\beta/T)(X_o(z)-X_p(z))+zTA_p(z). \tag{45}$$

Substituting (41)–(43) into (38), we have

$$X_p(z)=\frac{1}{z}((1-\alpha)X_p(z)+\alpha X_o(z))+\frac{T}{z}(zV_p(z)-TzA_p(z))+\frac{T^2}{2}A_p(z). \tag{46}$$

Substituting (44) and (45) into (46), the relationship between the predicted position and measured parameters is obtained as

$$\begin{aligned}
X_p(z) = {} & \frac{(\alpha+\beta)z-\alpha}{z^2+(\alpha+\beta-2)z+1-\alpha}X_o(z)\\
&+\frac{gz(z+1)}{2(z+\gamma-1)(z^2+(\alpha+\beta-2)z+1-\alpha)}T^2A_o(z). && (47)
\end{aligned}$$

Thus, the z-transform of the error $x_{\mathrm{o}k} - x_{\mathrm{p}k}$ is expressed as

$$\begin{aligned} E_{\mathrm{p}}(z) &= X_{\mathrm{o}}(z) - X_{\mathrm{p}}(z) \\ &= \frac{(z-1)^2}{z^2 + (\alpha+\beta-2)z + 1 - \alpha} X_{\mathrm{o}}(z) \\ &\quad - \frac{gz(z+1)}{2(z+\gamma-1)(z^2+(\alpha+\beta-2)z+1-\alpha)} T^2 A_{\mathrm{o}}(z). \end{aligned} \tag{48}$$

Here, the measured position and acceleration of a target with constant jerk J are:

$$x_{\mathrm{o}k} = J(kT)^3/6, \tag{49}$$

$$a_{\mathrm{o}k} = JkT, \tag{50}$$

and their z-transforms are:

$$X_{\mathrm{o}}(z) = \frac{z(z^2+4z+1)}{6(z-1)^4} JT^3, \tag{51}$$

$$A_{\mathrm{o}}(z) = \frac{z}{(z-1)^2} JT. \tag{52}$$

Substituting (51) and (52) into (48), we have

$$E_{\mathrm{p}}(z) = \frac{z(z^2 - 2\gamma z + 4z - \gamma + 1)}{6(z-1)(z+\gamma-1)(z^2+(\alpha+\beta-2)z-\alpha+1)} JT^3. \tag{53}$$

With the final value theorem $\lim_{z\to 1}(z-1)E_{\mathrm{p}}(z)$, we have $e_{\mathrm{fin,PAM}}$ ((16) of the main document).

Outline of derivation of $\sigma^2_{\mathrm{p,PAM}}$ and $e_{\mathrm{fin,PVAM}}$

The derivation of the performance indices of the PVAM filter is conducted using the same procedure as for the PAM filter. Because of this similarity and the complexity of the derivation process for the PVAM filter, this section provides only an outline of their derivation.

First, we consider the derivation of $\sigma^2_{\mathrm{p,PAM}}$. The linear system with respect to the variances and covariances of the smoothing parameters can be calculated using (1)–(3) and (7)–(9) under the assumptions of (14)–(19), (25), (26)–(28), (36), (37), and

$$E[(v_{\mathrm{o}k} - v_{\mathrm{t}k})^2] = B_{\mathrm{v}}, \tag{54}$$

$$E[(x_{\mathrm{o}k} - x_{\mathrm{t}k})(v_{\mathrm{o}k} - v_{\mathrm{t}k})] = 0, \tag{55}$$

$$E[(v_{\mathrm{o}k} - v_{\mathrm{t}k})(a_{\mathrm{o}k} - a_{\mathrm{t}k})] = 0. \tag{56}$$

The linear system is derived as:

$$\begin{aligned} &\alpha(2-\alpha)\sigma^2_{\mathrm{sx}} - (1-\alpha)^2(T^2\sigma^2_{\mathrm{sv}} + (T^4/4)\sigma^2_{\mathrm{sa}} + 2T\sigma^2_{\mathrm{sxv}} + T^2\sigma^2_{\mathrm{sxa}} + T^3\sigma^2_{\mathrm{sva}}) \\ &= \alpha^2 B_{\mathrm{x}}, \end{aligned} \tag{57}$$

$$\beta(2-\beta)\sigma^2_{\mathrm{sv}} - (1-\beta)^2(T^2\sigma^2_{\mathrm{sa}} + 2T\sigma^2_{\mathrm{sva}}) = \beta^2 B_{\mathrm{v}}, \tag{58}$$

$$\gamma(2-\gamma)\sigma_{\mathrm{sa}}^2 = \gamma^2 B_{\mathrm{a}}, \quad (59)$$

$$(\alpha-1)(\beta-1)T\sigma_{\mathrm{sv}}^2 + (1/2)(\alpha-1)(\beta-1)T^3\sigma_{\mathrm{sa}}^2 + (\alpha\beta-\alpha-\beta)\sigma_{\mathrm{sxv}}^2$$
$$+(\alpha-1)(\beta-1)T\sigma_{\mathrm{sxa}}^2 + (3/2)(\alpha-1)(\beta-1)T^2\sigma_{\mathrm{sva}}^2 = 0, \quad (60)$$

$$(1/2)(\alpha-1)(\gamma-1)T^2\sigma_{\mathrm{sa}}^2 + (\alpha\gamma-\alpha-\gamma)\sigma_{\mathrm{sxa}}^2 + (\alpha-1)(\gamma-1)T\sigma_{\mathrm{sva}}^2 = 0 \quad (61)$$

$$(\beta-1)(\gamma-1)T\sigma_{\mathrm{sa}}^2 + (\beta\gamma-\beta-\gamma)\sigma_{\mathrm{sva}}^2 = 0 \quad (62)$$

Substituting the solutions of the linear system involving (57)–(62) into (20), we have $\sigma_{\mathrm{p,PAM}}^2$ ((12) of the main document).

Next, we outline the derivation of $e_{\mathrm{fin,PVAM}}$. By applying a z-transform to (1)–(3) and (7)–(9) and their simplified forms, the predicted parameters in the z-domain are derived as:

$$A_{\mathrm{p}}(z) = \frac{\gamma}{z+\gamma-1}A_{\mathrm{o}}(z), \quad (63)$$

$$V_{\mathrm{p}}(z) = \frac{\beta}{z+\beta-1}V_{\mathrm{o}}(z) + \frac{\gamma z}{(z+\beta-1)(z+\gamma-1)}TA_{\mathrm{o}}(z), \quad (64)$$

$$X_{\mathrm{p}}(z) = \frac{\alpha}{z+\alpha-1}X_{\mathrm{o}}(z) + \frac{\beta z}{(z+\alpha-1)(z+\beta-1)}TV_{\mathrm{o}}(z)$$
$$+\frac{\gamma z(z-\beta+1)}{2(z+\alpha-1)(z+\beta-1)(z+\gamma-1)}T^2A_{\mathrm{o}}(z). \quad (65)$$

Here, the measured velocity of a target with constant jerk J is

$$v_{\mathrm{o}k} = J(kT)^2/2, \quad (66)$$

and its z-transform is:

$$V_{\mathrm{o}}(z) = \frac{z(z+1)}{2(z-1)^3}JT^2, \quad (67)$$

The z-transform of the predicted error for constant jerk target is derived as

$$E_{\mathrm{p}}(z) = \frac{z(z^2-2\beta z-2\gamma z+4z+\beta\gamma-\beta-\gamma+1)}{6(z-1)(z+\alpha-1)(z+\beta-1)(z+\gamma-1)}JT^3. \quad (68)$$

Applying the final value theorem to (68), we have $e_{\mathrm{fin,PVAM}}$ ((17) of the main document).
